# Reishi Protein LZ-8 Induces FOXP3^+^ Treg Expansion via a CD45-Dependent Signaling Pathway and Alleviates Acute Intestinal Inflammation in Mice

**DOI:** 10.1155/2013/513542

**Published:** 2013-06-24

**Authors:** Hsien-Yeh Hsu, Yen-Chou Kuan, Tung-Yi Lin, Shu-Ming Tsao, Jason Hsu, Li-Juan Ma, Fuu Sheu

**Affiliations:** ^1^Department of Biotechnology and Laboratory Science in Medicine, National Yang-Ming University, No. 155, Section 2, Li-Nong Street, Taipei 11221, Taiwan; ^2^The Genomics Research Center, Academia Sinica, Taipei 11574, Taiwan; ^3^Program in Molecular Medicine, National Yang-Ming University and Academia Sinica, Taipei 11574, Taiwan; ^4^Center for Biotechnology, National Taiwan University, No. 1, Section 4, Roosevelt Road, Taipei 10673, Taiwan; ^5^Department of Horticulture, National Taiwan University, No. 1, Section 4, Roosevelt Road, Taipei 10673, Taiwan; ^6^Fordham University, New York, NY 10458, USA

## Abstract

LZ-8, an immunomodulatory protein isolated from *Ganoderma lucidum* (also known as Ling-Zhi or Reishi), has been shown to promote cell proliferation and IL-2 production in T cells. In this study, we show that LZ-8 induces the expansion of both murine and human CD4^+^ T cells into FOXP3^+^ regulatory T (Treg) cells. LZ-8 treatment was found to stimulate a 4-fold and a 10-fold expansion in the Treg populations of murine and human primary CD4^+^ T cells, respectively. In addition, the expression of CTLA-4 and IL-10 was induced in LZ-8-treated CD4^+^ T cells. Using neutralizing antibodies and gene-deficient T-cell lines, we also found that LZ-8 promotes Treg expansion through a CD45-mediated signaling pathway and that the CD18-dependent induction of IL-2 was involved in Treg formation and IL-10 production. The suppressive activity of LZ-8 was confirmed using a murine model of DSS-induced colitis; the disease was alleviated by the adoptive transfer of LZ-8-treated CD4^+^ T cells. In conclusion, a new regulatory function for LZ-8 was identified, and the molecular mechanisms underlying this function were elucidated.

## 1. Introduction

*Ganoderma lucidum* (also known as Ling-Zhi or Reishi) is a medicinal mushroom that is widely appreciated as a traditional Chinese medicine throughout the world. It has been well documented that Reishi possesses a broad range of pharmacological properties including antitumor [1], immuno modulatory [2], and anti-inflammatory activities [3].

LZ-8 is an immunomodulatory protein that can be isolated from Reishi. LZ-8 was first discovered by Kino et al. [4], and its nucleotide sequence and structure have been characterized in several studies [5, 6]. LZ-8 activates murine effector T cells [2], activates antigen-presenting cells [7], stimulates cell proliferation and IL-2 production in human T cells [8], and promotes the maturation of human dendritic cells [9].

Regulatory T (Treg) cells are characterized by the expression of FOXP3, although other Treg subpopulations have been described, including IL-10-producing Tr1 cells, TGF-*β*-producing Th3 cells, and CD4^+^CD25^+^ T cells [10]. Tregs are indispensable for the control of pathogenic autoreactivity and the maintenance of immuno homeostasis. These cells exert their regulatory functions through various mechanisms, such as the secretion of IL-10 and TGF-*β*, the provision of inhibitory signals via cell-cell interactions, and altering the availability of factors required for immuno activation, such as IL-2 [11]. IL-2 is an immuno-potentiating cytokine that enhances T-cell survival, T-cell proliferation, and effector cell differentiation and has been termed the “T-cell growth factor.” Mice deficient in IL-2 signaling develop severe autoimmuno disease due to impaired Treg development [12–14], which confirms the importance of IL-2 for Treg differentiation and function.

Ulcerative colitis (UC) is a type of inflammatory bowel disease (IBD) that is characterized by leukocyte infiltration and the increased presence of inflammatory cytokines in the intestine, and DSS-induced colitis has been used as a model to study UC in mice. Oral ingestion of DSS causes a disruption in the intestinal barrier, which leads to intestinal inflammation that resembles the symptoms of UC. Tregs have been shown to play an essential role in the regulation of intestinal homeostasis, which has been confirmed by the fact that a genetic defect in Treg development can lead to a severe autoimmune response in the intestines [15].

We have previously demonstrated the mechanism by which LZ-8 stimulates IL-2 production in human T cells [8], and we have also suggested that LZ-8 has the potential to activate T-cell differentiation. In this study, we show that LZ-8 promotes Treg expansion, and we elucidate the underlying molecular mechanisms of this process. Furthermore, we also demonstrate the potential of LZ-8-treated T cells to attenuate intestinal inflammation using a murine model of DSS-induced colitis.

## 2. Materials and Methods

### 2.1. Reagents and Antibodies

Anti-human CD3 and CD28 antibodies, APC-conjugated anti-human CD25 antibody, PE-conjugated anti-human CTLA-4 antibody, and PE-conjugated anti-human FOXP3 staining sets were purchased from eBioscience (San Diego, CA, USA). Anti-human IL-2 and CD18 antibodies were purchased from Biolegend (San Diego, CA, USA). PHA was purchased from Sigma (St. Louis, MO, USA). The CFSE-staining kits were purchased from Molecular Probes (Eugene, OR, USA). Recombinant human IL-2 was purchased from ProSpec-Tany TechnoGene Ltd. (Israel). Phosphor-PLC*γ*I (T783) antibody was purchased from Stressgen Inc. (Ann Arbor, MI, USA). Phosphor-ZAP-70 (T319, T352) antibody was purchased from Cell Signaling Technologies (Danvers, MA, USA). Protein tyrosine phosphatase CD45 inhibitor was purchased from Calbiochem-Novabiochem Corp. (San Diego, CA, USA).

### 2.2. Preparation of Recombinant LZ-8 (rLZ-8)

The LZ-8 protein produced in *Saccharomyces cerevisiae* was prepared as described in our previous report [2]. Briefly, the LZ-8 gene (NCBI M58032.1) was cloned into the pYEX-S1 plasmid and transformed into *S. cerevisiae *DBY747. The LZ-8 was purified by fast protein liquid chromatography using a HiTrap Q column (Amersham Biosciences, Uppsala, Sweden). The purity of LZ-8 was >98% as analyzed by SDS-PAGE. The endotoxin level of purified LZ-8 was <0.012 EU/*μ*g as determined by the Limulus amoebocyte lysate assay.

### 2.3. Mice and Cell Cultures

Male BALB/c mice between 6 and 8 weeks of age were obtained from the National Laboratory Animal Center of Taiwan. All animal experiments and maintenance were performed according to the regulations set by the institutional animal care and use committee of National Yang-Ming University (approval number 951207). Mouse CD4^+^ T cells were purified from splenocytes by negative selection using magnetic beads (Miltenyi Biotech, Auburn, CA, USA). The cells were stimulated for 3 days with LZ-8 (1 *μ*g/mL) or anti-CD3 plus anti-CD28 mAbs (1 *μ*g/mL each), and these cells, together with nonstimulated control cells incubated for 3 days, were used in the following studies. For the adoptive transfer experiments, 1.5 × 10^6^ cells were injected intraperitoneally into each mouse. Primary human CD4^+^ T cells were purified from peripheral blood mononuclear cells (PBMC) by negative selection using magnetic beads. Jurkat, J.RT3-T3.5 (TCR-deficient) and J45.01 (CD45-deficient), human T-cell lines were obtained from the ATCC (Rockville, MD, USA), and the cells were cultured in RPMI 1640 medium supplemented with 10% fetal bovine serum and 2 mM l-glutamine (Life Technologies, Inc., MD, USA). Cells, including control cells, were stimulated with either LZ-8 (1 *μ*g/mL) or anti-CD3 plus anti-CD28 mAbs (1 *μ*g/mL each), and these cells, in addition to unstimulated cells, were then incubated for growth. In some experiments, the cells were pretreated with the indicated reagents for 1 h prior to stimulation. All cells were cultured in flat-bottom 24-well culture plates at a density of 1 × 10^6^ cells/mL at 37°C in a humidified atmosphere supplemented with 5% CO_2_.

### 2.4. Induction of Colitis and the Assessment of Colonic Damage

Male BALB/c mice between 6 and 8 weeks of age were weighed and randomly distributed into five groups (*n* = 5). Colitis was induced by adding 4% (w/v) DSS to the drinking water for 7 days. One group that was administered normal drinking water served as the control group. Intraperitoneal injections of CD4^+^ T cells or PBS as a vehicle were carried out 1 day before DSS induction. The body weights of each mouse were recorded, and the mice were sacrificed on day 7. The colons were removed and rinsed with ice-cold PBS. Segments (0.5 cm in length) of the distal colon were removed, fixed with 10% formalin, and stained with hematoxylin and eosin for the histopathological analysis.

### 2.5. Analysis of Treg Expansion and Functioning

Treg expansion was analyzed by flow cytometry, and the data are presented as the percent of positive fluorescent cells present among the total cells. In brief, the cells were harvested and resuspended in ice-cold PBS, and the expression of CD25, CTLA4, and FOXP3 was detected using fluorescence-labeled mAb. Cell-free supernatants were collected to determine IL-2 and IL-10 production using ELISA kits (R&D, Minneapolis, MN, USA), according to the manufacturer's protocols.

### 2.6. Mix Leukocytes Reaction

For the suppressor cells, human CD4^+^ T cells were stimulated with LZ-8 (1 *μ*g/mL), anti-CD3 plus anti-CD28 mAbs (1 *μ*g/mL each), or without stimulation for 3 days. For the responder cells, human CD4^+^ cells were labeled with 1 *μ*M CFSE following the manufacturer's protocol and then cultured with PHA (2 *μ*g/mL) for 4 days in the presence or absence of suppressers. The suppressor to responder ratio was 1 : 3 (2.5 × 10^5^ : 7.5 × 10^5^). The cell division was monitored by flow cytometry.

### 2.7. Analysis of Protein Phosphorylation by Western Blotting

These experiments were carried out as described previously [8].

### 2.8. Statistical Analysis

Significant differences between the experimental and control groups were examined by an analysis of variance, and the statistical significance was set at *P* < 0.05. The data are representative of at least three independent experiments and are expressed as the mean ± SD.

## 3. Results

### 3.1. LZ-8 Stimulates Treg Expansion in Murine and Human CD4^+^ T Cells

**Figure 1:**
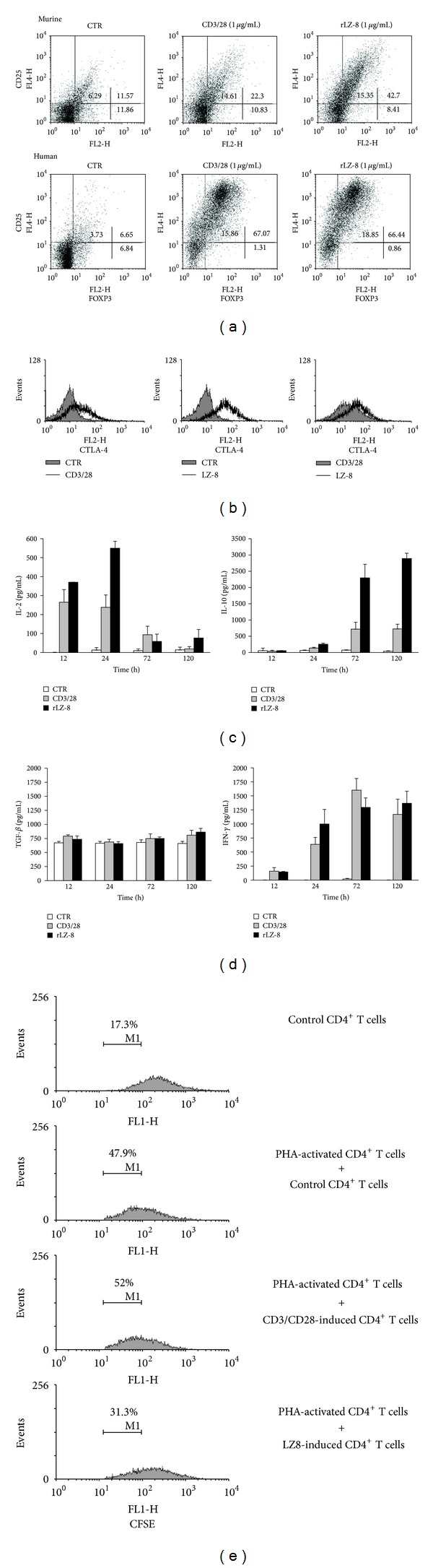
LZ-8 stimulates Treg expansion in murine and human CD4^+^ T cells. Primary murine and human CD4^+^ T cells were cultured for 72 h, either in the absence of stimulation as the control (CTR), in the presence of stimulation with recombinant LZ-8 antibody (rLZ-8; 1 *μ*g/mL), or in the presence of stimulation with anti-CD3/28 antibodies (CD3/CD28; 1 *μ*g/mL each, (a, b). The cells were then harvested for flow cytometry analysis of CD25, FOXP3, and CTLA-4 expression. (c and d) The levels of IL-2, IL-10, IFN-*γ*, and TGF-*β* in the culture supernatants were measured by ELISA. (e) The cell proliferation of CD4^+^ T cells treated with or without PHA (2 *μ*g/mL) and then cocultured with indicated CD4^+^ T cells was monitored by cell-trace CFSE fluorescence via flow cytometry.

Although it has been shown that LZ-8 stimulates an immuno response by activating T cells and monocytes, it remains unknown whether LZ-8 may have an immuno suppressive function. To address this question, primary murine and human CD4^+^ T cells were cultured with LZ-8 (1 *μ*g/mL) for 72 h, and Treg cell expansion was examined using flow cytometry. LZ-8 treatment expanded the population of CD25^+^FOXP3^+^ Treg by approximately 4-fold and 10-fold when using murine and human CD4^+^ T cells, respectively, (Figure 1(a)). The expression of CTLA-4, an immuno suppressive surface molecule, on LZ-8-treated CD4^+^ T cells was also upregulated following treatment (Figure 1(b)). Interestingly, a time-course study of secreted cytokines in the culture supernatants revealed that LZ-8 stimulated IL-2 production at 24 h, which was followed by enhanced IL-10 production at 120 h (Figure 1(c)). The cells treated with CD3/28 antibodies also showed a significant increase in Treg expansion, as well as increased expression of CTLA-4, IL-2, and IL-10 (Figure 1).

The TGF-*β* presented in the culture supernatant was also examined. However, neither LZ-8 nor CD3/CD28 treatment stimulated TGF-*β* production (Figure 1(d)). Notably, we also discovered that as comparable to CD3/CD28 stimulation, LZ-8 induced IFN-*γ* secretion by CD4^+^ T cells (Figure 1(d)). To test whether the effect of LZ-8-induced CD4^+^ T cells was immuno-enhancing or immuno-suppressive, we conducted a mix leukocytes reaction. LZ-8-induced CD4^+^ T cells were able to inhibit PHA-induced T-cell proliferation by 40%; however, the cell proliferation rates were similar in the cells that were cocultured with the control and the CD3/CD28-induced T cells (Figure 1(e)). Collectively we confirmed that LZ-8 was capable of inducing the expansion of IL-2 and IL-10-expressing Tregs and that the LZ-8-induced CD4^+^ T cells had regulatory activity.

### 3.2. CD18-Dependent IL-2 Production Is Crucial for LZ-8-Induced Treg Expansion

**Figure 2:**
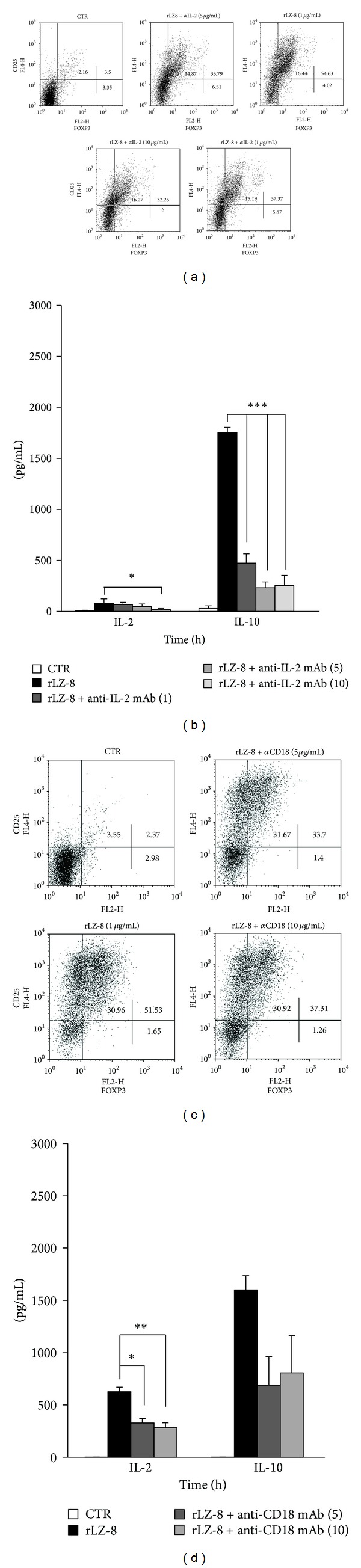
CD18-dependent IL-2 production is crucial for LZ-8-induced Treg expansion. Primary human CD4^+^ T cells were pretreated with or without (a, b) anti-IL-2 antibodies (*α*IL-2; 1, 5, 10 *μ*g/mL) or (c, d) anti-CD18 antibodies (5, 10 *μ*g/mL) for one hour prior to rLZ-8 stimulation for 72 h. (a, c) The cells were then harvested for flow cytometry analysis of CD25 and FOXP3 expression. (b, d) The levels of IL-2 and IL-10 in the culture supernatants were measured by ELISA. The asterisks indicate significant differences (*P < 0.05; **P < 0.01; ***P < 0.001) between the different treatments.

IL-2 has the capacity to promote T-cell differentiation [12–14], and our time-course cytokine study also indicated that there was IL-2 consumption during Treg expansion induced by LZ-8 (Figure 1(c)). To determine whether IL-2 is involved in LZ-8-induced Treg expansion, we neutralized IL-2 by applying anti-IL-2 antibodies (*α*IL-2; 1, 5, 10 *μ*g/mL) 1 h prior to LZ-8 stimulation. As expected, *α*IL-2 completely blocked IL-2 secretion (Figure 2(b)). Notably, *α*IL-2 also significantly blocked LZ-8-induced Treg expansion (Figure 2(a)) and IL-10 production (Figure 2(b)) in a dose-dependent manner. It has been reported that LZ-8 enhances expression of ICAM-1 [16], a ligand of the integrin CD18, and we discovered that blocking CD18 with neutralizing antibodies 1 h prior to LZ-8 stimulation significantly reduced IL-2 production (Figure 2(d)). This blockade of the CD18 pathway also decreased LZ-8-induced Treg development and IL-10 production (Figures 2(c) and 2(d)). Taken together, these findings suggested that CD18 signaling is involved in LZ-8-induced IL-2 production, highlighting the indispensable role of IL-2 in LZ-8-induced Treg expansion.

### 3.3. LZ-8 Promotes Treg Expansion through a CD45-Mediated Signal Pathway

**Figure 3:**
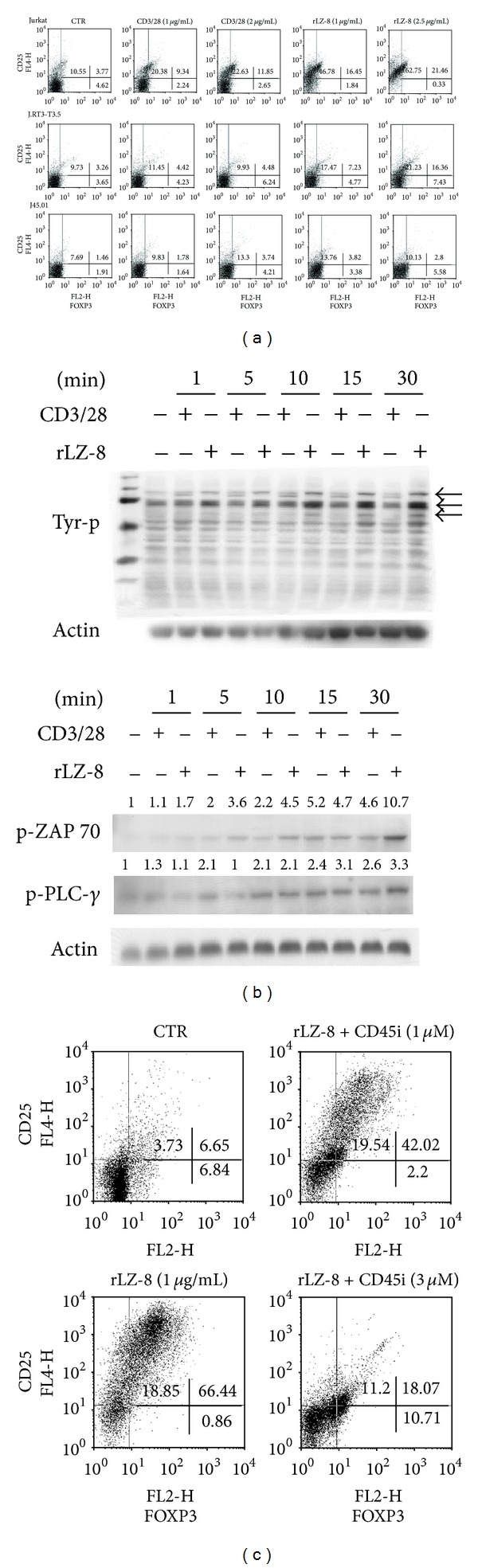
LZ-8 promotes Treg expansion via a CD45-mediated signal pathway. (a) Human T-cell Jurkat (WT), J.RT3-T3.5 (TCR^−/−^), and J45.01 (CD45^−/−^) cells were cultured without stimulation as the control (CTR), with stimulation by rLZ-8 (1, 2 *μ*g/mL), or with stimulation by anti-CD3/28 antibodies (CD3/CD28; 1,  2 *μ*g/mL) for 72 h. The cells were then harvested for flow cytometry analysis of CD25 and FOXP3 expression. (b) J.RT3-T3.5 cells were stimulated with or without rLZ-8 (2 *μ*g/mL) or with or without anti-CD3/28 antibodies (CD3/28; 2 *μ*g/mL) for the indicated time periods. The total cell lysates were collected for protein phosphorylation analysis. (c) Primary human CD4^+^ T cells were pretreated with or without CD45 phosphatase inhibitor (CD45i; 1, 3 *μ*M) for one hour prior to rLZ-8 stimulation for 72 h. The cells were then harvested for flow cytometry analysis of CD25 and FOXP3 expression.

Despite the fact that many studies have shown the capacity of LZ-8 to stimulate CD4^+^ T cells, the interaction between LZ-8 and T cells remains unclear. Therefore, we investigated the potential receptor for LZ-8 on human CD4^+^ T cells using various T-cell lines, namely, Jurkat (WT), J.RT3-T3.5 (TCR^−/−^), and J45.01 (CD45^−/−^) cells. The stimulation of Jurkat cells with LZ-8 and anti-CD3/28 antibodies (*α*CD3/28; 1 *μ*g/mL each) expanded the population of Tregs (Figure 3(a)). As expected, *α*CD3/28 stimulation failed to induce Treg expansion in TCR^−/−^  J.RT3-T3.5 cells, whereas LZ-8 continued to induce Treg expansion in TCR^−/−^ cells (Figure 3(a)). These results suggest that the TCR is not the receptor of LZ-8. Notably, it has been shown that LZ-8 fails to induce a significant increase in Treg expansion in CD45^−/−^ J45.01 cells (Figure 3(a)), which indicates that CD45 is likely the primary receptor that mediates the interactions between human CD4^+^ T cells and LZ-8. To elucidate the pathway downstream of CD45, J.RT3-T3.5 cells were stimulated with LZ-8, harvested, and then lysed for immunoblot analysis. LZ-8 stimulation increased the amount of phosphorylated Zap-70 and phospholipase C-*γ* (Figure 3(b)), indicating that LZ-8 activates human T cells via CD45-dependent activation of T-cell proximal signaling. This finding was confirmed using a CD45 inhibitor (CD45i; 1, 3 *μ*M), where the application of CD45i significantly reduced LZ-8-induced Treg expansion (Figure 3(c)). Taken as a whole, these findings demonstrate that LZ-8-induced Treg expansion occurs via CD45-mediated T-cell proximal signaling.

### 3.4. Adoptive Transfer of LZ-8-Stimulated CD4^+^ T Cells Alleviates DSS-Induced Colitis

**Figure 4:**
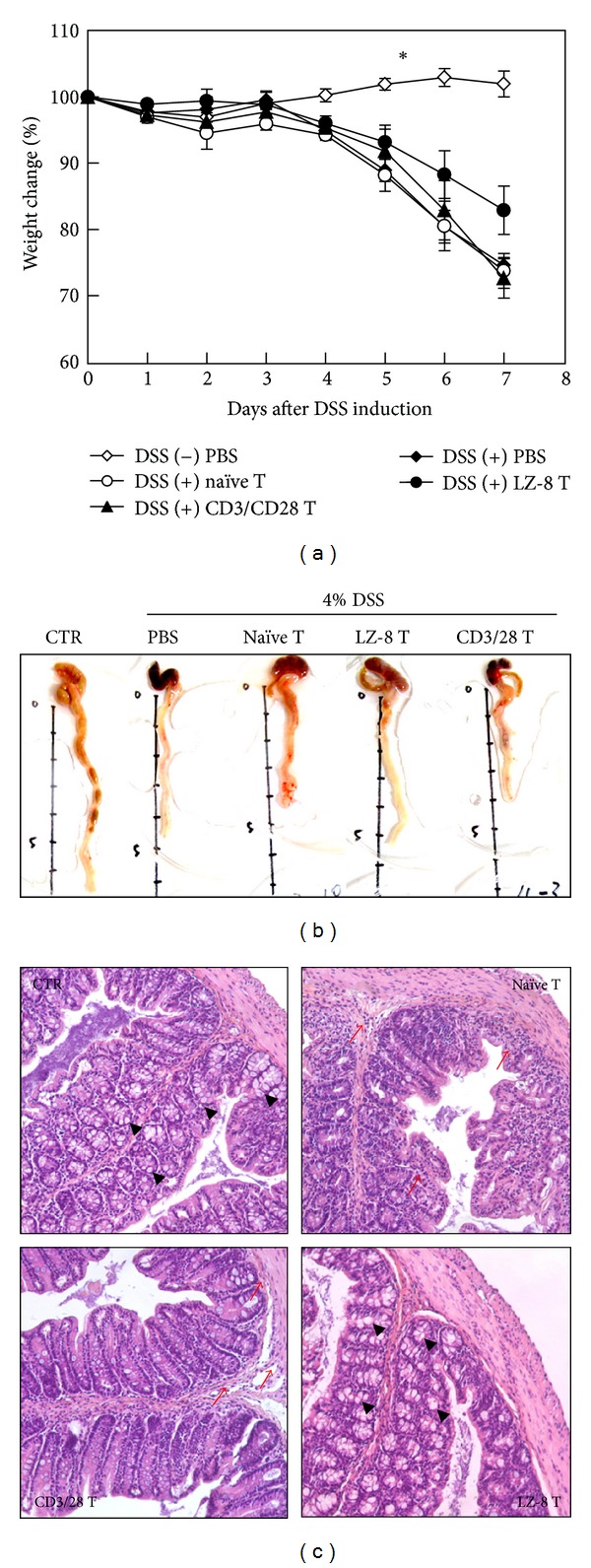
Adoptive transfer of LZ-8-stimulated CD4^+^ T cells alleviates DSS-induced colitis. Primary murine CD4^+^ T cells were cultured either without stimulation (naïve T), with rLZ-8 (LZ-8 T; 1 *μ*g/mL), or with anti-CD3/28 antibodies (CD3/28 T; 1 *μ*g/mL each) for 72 h. Colitis was induced by adding 4% DSS to the normal drinking water, with one group of mice receiving normal drinking water as a control (CTR). Intraperitoneal injections of PBS (200 *μ*L/mouse), naïve T cells, LZ-8 T cells, or CD3/28 T cells (1.5 × 10^7^ cells suspended in 200 *μ*L PBS/mouse) were carried out 24 h prior to DSS induction. (a) The percentage of weight change in each mouse was monitored daily, and the data are presented as the mean ± SD. The asterisks indicate significant differences among DSS-treated groups (*P* < 0.05, *n* = 5). (b, c) Colons from each mouse were excised for pathological (b) and histological (c) examination seven days after DSS induction. Representative specimens from five animals are presented. (c) The infiltrating leukocytes and intact goblet cells are highlighted by arrows and arrowheads, respectively.

To evaluate whether LZ-8-induced Tregs possess immuno suppressive capabilities, we employed a murine model of DSS-induced acute colitis as described in Section 2.4. The weight loss of mice receiving LZ-8-stimulated CD4^+^ T cells (T_LZ-8_) was less severe than that observed in other DSS-treated mice and was significantly lower than that of mice receiving naïve CD4^+^ T cells (T_naïve_; Figure 4(a)). Furthermore, colon samples from mice receiving T_LZ-8_ showed the greatest similarity to those of control mice (Figure 4(b)). Reductions in colon length among mice receiving PBS, T_naïve_ cells, or *α*CD3/28-stimulated CD4^+^ T (T_*α*CD3/28_) cells were also observed (Figure 4(b)). Moreover, during the histological analysis, a normal colon structure and normal crypt morphology were observed in the control mice (Figure 4(c), upper left, UL), whereas leukocyte infiltration and goblet cell depletion (as indicated by the arrows and arrowheads, respectively) were observed in the DSS-treated mice. In addition, the adoptive transfer of T_LZ-8_ (Figure 4(c), LR), but not of T_naïve_ (Figure 4(c), UR) or T_*α*CD3/28_ (Figure 4(c), LL), significantly reduced leukocyte infiltration and goblet cell depletion.

## 4. Discussion

The immunomodulatory activities of LZ-8 with regard to immuno cells have been extensively studied [2, 7–9]; however, few reports have explored the immuno suppressive activity or regulatory functions of LZ-8. In our previous research, we reported that LZ-8 directly induced the activation and IL-2 production of murine and human CD4^+^ T cells in the absence of antigen-presenting cells [2, 8]. In this study, using both murine and human CD4^+^ T cells, we found that LZ-8 expanded CD25^+^FOXP3^+^ Tregs (Figure 1(a)). We also noticed that *α*CD3/28 stimulation expanded the proportion of CD25^+^FOXP3^+^ Treg cells (Figure 1(a)). These findings are in agreement with those of Chen et al., who demonstrated that the application of a superagonistic anti-CD28 antibody (supCD28mAb, D665) expanded FOXP3^+^ regulatory T cells [17]. However, the anti-CD28 antibody used in this study was a different clone (CD28.6, eBioscience Cat. No. 16-0288). In addition, we showed that LZ-8-induced CD4^+^ T cells expressed a higher level of CTLA-4 (Figure 1(b)), a key molecule involved in controlling regulatory T-cell function. Furthermore, this effect corresponded to the expansion of Tregs and the colitis suppressive effect of LZ-8-stimulated CD4^+^ T cells (Figure 4).

Notably, we discovered that LZ-8-induced CD4^+^ T cells were also able to secrete IFN-*γ*, a proinflammatory cytokine (Figure 1(d)). This showed that although LZ-8 expanded the CD25^+^FOXP3^+^ Treg population, other effector cells that lie within the population might also be expanded. It has been reported that a certain subpopulation of Helios^−^ cells were observed to secret effector cytokines such as the IFN-*γ* [18]. Therefore, in order to specify the subtype of LZ-8-induced CD4^+^ T cells, further examinations on surface markers such as the Helios and the CD127 should be carried out in the future. Nevertheless, using a mix leukocytes reaction, we demonstrated the immuno regulatory activity of the LZ-8-induced CD4^+^ T cell (Figure 1(e)).

It has been previously demonstrated that FOXP3^+^ thymocytes are dramatically reduced in IL-2/IL-2R deficient mice and that neutralization of IL-2 using *α*IL-2 monoclonal antibody results in autoimmuno gastritis in mice [12–14]. These findings highlight the critical role of IL-2 signaling in the development and functioning of regulatory T cells. In the present study, we demonstrated that LZ-8-induced IL-2 production was required for LZ-8-induced Treg expansion and functioning. Furthermore, it has been reported that CD18 signaling is crucial for the developmental and suppressive functions of both innate and peripherally induced Tregs [19]. We found that blocking CD18 signaling resulted in a significant reduction in IL-2 production and reduced Treg expansion among LZ-8-induced CD4^+^ T cells (Figure 2). Taken together, we propose that CD18-mediated IL-2 production is critical to LZ-8-induced Treg expansion. Although it has been previously reported that LZ-8 induces T-cell activation [2, 7–9], the molecular receptor responsible for LZ-8-mediated T-cell activation remains unknown. In this study, we demonstrated using gene knockout and inhibition studies that CD45 appears to act as a major downstream molecule for LZ-8 in human T cells (Figure 3). Finally, in this context, we also demonstrated that LZ-8 activates T cells through a CD45-dependent T-cell proximal signaling pathway.

FOXP3^+^ cells, CD4^+^CD25^+^FOXP3^+^ cells, and IL-10-secreting CD4^+^CD25^+^ T cells all play key roles in the regulation and suppression of DSS-induced colitis [20, 21]. In addition, adoptive transfer of Tregs was shown to prevent the development of colitis and has previously been shown to attenuate established IBD [22, 23] Thus, therapies targeting Tregs should provide promising insights for the treatment of IBD and are under intense investigation [24, 25]. We have shown here that LZ-8-induced CD4^+^ T cells alleviates colitis (Figure 4), and it has previously been shown that LZ-8 possesses immunosuppressive activity and can prevent insulitis in nonobese diabetic mice and prolong allograft survival [26, 27]. Thus, our results suggest that these suppressive effects may be a result of Treg expansion. Nonetheless, although we have demonstrated the potential of LZ-8-induced CD4^+^ T cell on ameliorating mice colitis, further experiments are required to define the effect and action mechanism of LZ-8 as an immuno therapeutic agent.

In conclusion, we showed that LZ-8 induces Treg expansion and that LZ-8-induced Treg expansion is dependent on CD18-dependent IL-2 production; furthermore, this induction seemed to occur via CD45-mediated T-cell proximal signaling. In addition, we demonstrated that LZ-8-induced Tregs were capable of alleviating acute colitis. Thus, these studies provide new insights into the immuno modulatory mechanism(s) of LZ-8 and suggest that LZ-8 has the potential to help control intestinal inflammation.
